# Palladium Nanoparticles Fabricated by Green Chemistry: Promising Chemotherapeutic, Antioxidant and Antimicrobial Agents

**DOI:** 10.3390/ma13173661

**Published:** 2020-08-19

**Authors:** Sherif Ashraf Fahmy, Eduard Preis, Udo Bakowsky, Hassan Mohamed El-Said Azzazy

**Affiliations:** 1 Department of Chemistry, School of Sciences & Engineering, The American University in Cairo, AUC Avenue, P.O. Box 74, New Cairo 11835, Egypt; sheriffahmy@aucegypt.edu; 2 Department of Pharmaceutics and Biopharmaceutics, University of Marburg, Robert-Koch-Str. 4, 35037 Marburg, Germany; eduard.preis@pharmazie.uni-marburg.de

**Keywords:** green synthesis, plant extracts, palladium nanoparticles, anticancer activity, antibacterial activity, antioxidants

## Abstract

Palladium nanoparticles (Pd NPs) showed great potential in biomedical applications because of their unique physicochemical properties. Various conventional physical and chemical methods have been used for the synthesis of Pd NPs. However, these methods include the use of hazardous reagents and reaction conditions, which may be toxic to health and to the environment. Thus, eco-friendly, rapid, and economic approaches for the synthesis of Pd NPs have been developed. Bacteria, fungi, yeast, seaweeds, plants, and plant extracts were used to prepare Pd NPs. This review highlights the most recent studies for the biosynthesis of Pd NPs, factors controlling their synthesis, and their potential biomedical applications.

## 1. Introduction

Palladium nanoparticles (Pd NPs) have significant chemical and thermal stabilities, electronic properties, and optical properties [1,2]. They could also be biofunctionalized to enable their medical applications [3,4]. Moreover, Pd NPs were used as photothermal agents, drug carriers, and prodrug activators. They possess antimicrobial, antioxidant, and cytotoxic activities [2,5,6]. Several studies reported on the synthesis of Pd NPs using hazardous, expensive, and multistep conventional techniques such as sol-gel, chemical reduction, and electrochemical and chemical precipitation [1,2].

However, in the past few years, there has been a growing interest in utilizing green chemistry in the production of Pd NPs using eco-friendly and biocompatible bio-derived products, including microorganisms (such as bacteria, viruses, fungi, and yeast) and seaweeds [7,8,9,10,11,12,13,14,15,16,17,18,19,20,21]. In addition to these bio-derived products, utilizing plant extracts in the biosynthesis of Pd NPs (phytosynthesis) was suggested to be advantageous over other bio-derived materials. Unlike microorganisms and seaweeds, using plant extracts for the biosynthesis of Pd NPs is simple, rapid, and cost-effective. Furthermore, the phytosynthesis process can easily be optimized for large-scale production of more stable Pd NPs. On the other hand, plant extracts harbor various bioactive metabolites that could act as natural reducing and capping agents during the phytosynthesis of Pd NPs [22,23,24,25]. To achieve phytosynthesis, sonication, microwave, magnetic, and hydrothermal methods are employed along with non-hazardous solvents and lower temperatures [7]. This review covers the state-of-the-art in green synthesis of Pd NPs as well as their biomedical applications.

## 2. Green Synthesis of Pd NPs

Microorganisms (MOs), algae, and plant extracts were used for the bioreduction of metals [26,27,28]. The biosynthesis of Pd NPs is a safe, clean, and eco-friendly approach to produce NPs of versatile sizes, shapes, physical, chemical, and biological properties [29].

The use of MOs (specially fungi) in the green synthesis is promising because they are abundant and can bio-reduce Pd ions via enzymatic activity, converting them into Pd atoms while maintaining proper control over the sizes of the NPs through bio-patterning [30,31]. However, the use of MOs in the green synthesis involves a sophisticated and time-consuming multistep process. The localized heating (employing microwave heat, for instance) needed for the NPs to be created could deform the MOs enzymes required for the bioreduction to take place [29,32]. Additionally, many aspects should be considered in the process of producing Pd NPs using MOs, such as the careful selection of the most appropriate MO because each one interacts differently with metal ions according to the intrinsic enzymatic activity and biochemical processes. Additionally, culturing methods and conditions (such as light, temperature, pH, inoculation, and nutrients) are very crucial and should be optimized. A sterile environment should be maintained to prevent contamination with other MOs. Finally, reaction conditions should be controlled upon mixing the MO with the metal ions to obtain a large scale and stable Pd NPs with well-defined size and morphology [7,8,9,10,11,12,13,14,15,16,17,18,19,20,21]. Hence, the use of MO suffers many drawbacks, which makes its employment in the green synthesis of Pd NPs challenging.

Algae have recently been reported in the biosynthesis of Pd NPs [33]. They are rich in broad-spectrum bioactive compounds and natural reducing agents, making them very promising in the biogenic synthesis of Pd NPs. Additionally, they are easily harvested, scalable, and their surfaces possess negative charges facilitating the nucleation and growth of the nanoparticles. Hence, algae can be used for low-cost large-scale production of Pd NPs. Various types of algae, including red, brown, and green algae, were used for biosynthesis and tailoring of Pd NPs [33,34].

Contrary to MOs, the use of plant extracts in the biosynthesis of Pd NPs has attracted much attention in the past few years because of their advantages over other biological entities. The green synthesis of Pd NPs using plant extracts is a single-step process that does not require the elaborate steps of isolation, culture optimization, and maintenance [7,8,9,10,11,12,13,14,15,16,17,18,19,20,21,22,23]. Moreover, the use of plant extracts is cost-effective, safe, rapid, and is easier to scale up. Additionally, the Pd NPs produced via phytosynthesis are more stable and are created at faster rates compared to MO. Plants are rich in various bioactive secondary metabolites and organic reducing agents such as phenols, flavonoids, alkaloids, saponins, vitamins, aldehydes, and steroids [8,9,35,36,37,38]. These compounds play the primary role in the phytosynthesis of the Pd NPs via bioreduction of the metal ions and stabilizing the resulted Pd NPs [8,9]. Hence, many studies reported the use of plants and plant extracts for the rapid biosynthesis of various stable Pd NPs using environmentally friendly approaches [39,40,41].

Some plants also possess therapeutic actions such as antimicrobial, anticancer, or antioxidant activities. Hence, they impart a synergistic therapeutic effect upon their combination with Pd NPs [24,25].

## 3. Mechanism of Biogenic Synthesis of Pd NPs

**Figure 1:**
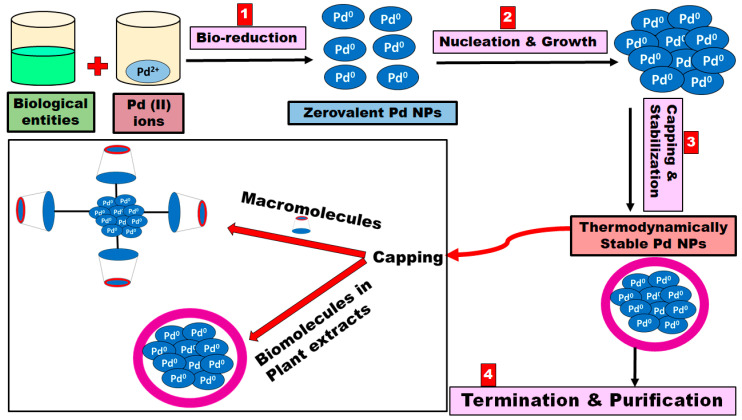
Schematic diagram representing the four-step mechanism for the biosynthesis of Pd NPs.

The biosynthesis of the Pd NPs generally involves mixing the metal salt solutions with the plant extract at room temperature, and the nanoparticles are created rapidly and indicated by the change in color of the reaction medium [42]. However, many parameters should be optimized to control the shape, size, and crystallinity of Pd NPs, including the origin of the biological extract and its concentration, metal salt concentration, the temperature of the reaction media, pH of the reaction media, rate at which extract is added, and the contact time [43,44,45,46,47]. While the exact mechanism of the biosynthesis of Pd NPs are not well known and more studies are required, a fundamental bottom-up mechanism involving four major steps is suggested (Figure 1) [48,49]. The initial activation step in the biosynthesis of Pd NPs is the bioreduction and nucleation of the metal ions at which the metal ions are reduced into their zero-oxidation states [50]. The second step involves the subsequent growth and agglomeration of the small Pd NPs into larger and more thermodynamically stable Pd NPs. Then, a termination step occurs where a variety of Pd NPs shapes are created, depending on the optimized conditions, such as spheres, rods, triangles, wires, pentagons, or hexagons [50,51]. During this phase, stabilization of the Pd NPs is undertaken via the plant extract, which contains various functional groups that could act as capping agents such as alcohols, aldehydes, amines, carboxylic acid, and ketones [50]. Finally, the biosynthesized Pd NPs are purified and washed via centrifugation [51]. It is worth mentioning that few recent studies reported on the use of supramolecules (macromolecules) such as calix[n]arenes and cyclodextrins as potential stabilizing agents for the biosynthesized Pd NPs [52,53,54,55,56]. They have been chosen as promising capping agents due to their biocompatible and eco-friendly nature [57]. Macromolecules could act as capping agents via surface attachment on the targeted Pd NPs through a combination of hydrophobic, charge transfer, hydrogen bonding, covalent bonding, or ion-dipole interactions (Figure 1) [58,59,60].

## 4. Phytosynthesis of Pd NPs

**Figure 2:**
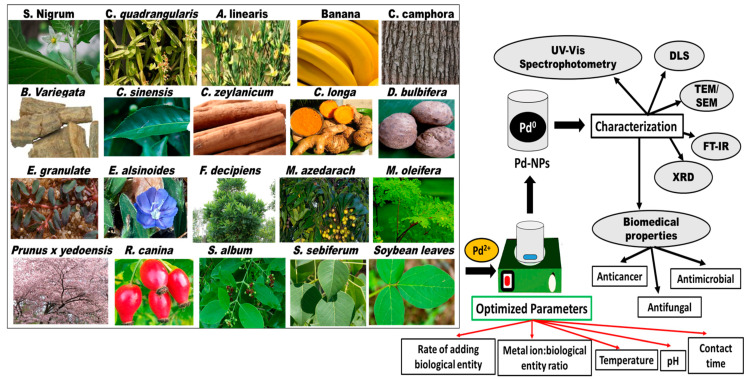
Schematic diagram summarizing the plant species employed in the bioreduction of Pd NPs, the parameters to be optimized, and the characterization of the fabricated nanoparticles.

Pd NPs are biosynthesized using various plant species, and the sizes, shapes, monodispersity, yield, and crystallinity of the fabricated nanoparticles are controlled by optimizing the synthesis conditions (Figure 2 and Table 1) [61,62]. The prepared Pd NPs were characterized using UV/Vis spectrophotometry, dynamic light scattering (DLS), scanning electron microscopy (SEM), transmission electron microscopy (TEM), Fourier transform infrared spectroscopy (FTIR), and powder X-ray diffraction (XRD). Spectroscopic and diffractographic techniques play a crucial role in the identification and characterization of Pd NPs [61,62].

Phytosynthesis of spherical Pd NPs with an average size of 21.55 nm was performed using *Solanum nigrum* leaf extract [25]. The palladium (II) ions were stirred with the leaf extract for 10 min at room temperature. The disappearance of the UV/Vis absorption spectrum at a peak range of 370–440 nm corresponding to Pd (II) ions suggested the reduction of Pd (II) [25]. The biosynthesized Pd NPs exhibited significant bactericidal activity against *Escherichia coli* (zone of inhibition: 18 and 19 mm, respectively) [25]. Phytosynthesis of Pd NPs using *Cissus quadrangularis* stem extract involved the same preparation conditions [63]. The biosynthesized nanoparticles were spherical with an average size range of 12–26 nm. The formation of Pd NPs was confirmed by the disappearance of the UV/Vis absorption spectrum at 420 nm, corresponding to Pd (II) ions [63]. Pd NPs prepared by *Cissus quadrangularis* stem extract also showed remarkable antibacterial activity against *E. coli* at certain concentrations [63]. In another study, *Aspalathus linearis* leaf extract was stirred with the Pd (II) ions for 30 min at room temperature, then dried [64]. The formation of Pd NPs was initially confirmed qualitatively by the conversion of the initial solution color from light yellow to dark brown. The disappearance of the sharp UV/Vis absorption peak at 284.5 nm corresponding to *Aspalathus linearis* leaf extract and the appearance of an absorption continuum, characteristic for the reduced Pd NPs because of the surface plasmon resonance, confirmed the formation of the nanoparticles [64]. TEM investigations showed spherical, triangular, rectangular, cubic, and decahedron nanoparticles with an average size of 12.7 nm [64].

Vaghela et al. [65] reported the biosynthesis of Pd NPs using *Bauhinia variegate* bark extract. Cylindrical Pd NPs were observed with average size from 2 to 9 nm after hot centrifugation at 6000 rpm for 10–15 min [65]. The disappearance of the UV/Vis absorption spectrum observed at 420 nm, corresponding to Pd (II) ions, confirmed the generation of Pd NPs [65]. The phytosynthesized Pd NPs have shown to have potent antibacterial activity against the Gram-positive bacteria, *Bacillus subtilis,* and antifungal activity against *Candida albicans.* The nanoparticles have also shown potent cytotoxic activity against MCF-7 breast cancer cells (IC50: 18.34 µg/mL) [65]. Lebasch et al. [66] developed an eco-friendly and facile method to biosynthesize Pd NPs using *Camellia sinensis* leaves extract. The biosynthesized Pd NPs, having an average size of 7 nm and spherical morphology, were prepared by the dropwise addition of leaves extract to Pd (II) ions solution (1:10) and heating at 100 °C for 1 h [66]. The disappearance of the UV/Vis absorption spectrum observed at 400 nm corresponding to Pd (II) ions suggested the reduction of Pd (II) ions and the creation of Pd NPs [66]. Azizi et al. [6] reported the phytosynthesis of spherical Pd NPs with an average size range of 6–18 nm, mediated by *Camellia sinensis* (white tea) extract [6]. The palladium (II) ions were continuously stirred with the white tea extract (1:1) for 2 h at 40 °C and the pH was found to be decreased from 7.5 to 5.6 (after the reaction). The typical peak of Pd (II) ions observed at 410 nm disappeared after the bioreduction accompanied by the appearance of a broad absorption continuum indicating the production of the Pd NPs [6]. The biosynthesized nanoparticles showed significant antioxidant effect and bactericidal activity against *Staphylococcus epidermidis* and *Escherichia coli* at minimum inhibitory concentrations of 0.15 µM and 0.313 µM (zone of inhibition: 17 and 14 mm, respectively) [6]. Moreover, the Pd NPs mediated by white tea was found to possess a more potent anticancer activity toward human leukemia (MOLT-4) cells compared to cisplatin and doxorubicin (IC50: 0.006 µM, 0.894 µM, and 2.133 µM, respectively), and no cytotoxic effect was observed on the normal human fibroblasts [6]. Sathishkumar et al. [67] obtained crystalline Pd NPs of 15–20 nm average size range using *Cinnamomum zeylanicum* bark extract, which was incubated with the Pd (II) ions solution in a rotary shaker at 160 rpm in the dark at 30 °C and pH > 5 for 72 h [67]. The same procedure was used to produce spherical Pd NPs mediated by *Curcuma longa* tuber extract of an average size range of 10–15 nm [68]. In another study, *Cinnamomum camphora* leaf broth was used to prepare quasi-spherical and irregular Pd NPs of an average size of 3.2–6 nm [69]. The leaf broth was incubated in the dark with the Pd (II) ions solution in a rotary shaker at 150 rpm in the dark at 30 °C for 12 h [69]. The formation of Pd NPs mediated by *Cinnamomum camphora* leaf broth was confirmed by the disappearance of the original UV/Vis peak of Pd (II) ions, observed above 300 nm, and the appearance of new broad absorption continuum in the visible-near-ultraviolet region [69]. Nadagouda et al. [70] developed a simple method for the bulk green production of Pd NPs using coffee and tea powder extracts. The spherical nanoparticles with size range of 5–100 nm were prepared by simple mixing of the metal ions with the powder extracts and confirmed by the appearance of a characteristic broad UV/Vis continuum in the range of 200–1200 nm [70]. Ghosh et al. [71] employed the tuber extract of *Dioscorea bulbifera* to generate spherical and blunt-ended cubical Pd NPs of an average size range of 10–25 nm by conducting the bio-reaction at 100 °C for 5 h [71]. The UV/Vis observation showed disappearance of peak at 420 nm, particularly to Pd (II) ions and higher absorbance compared to Pd NPs alone, indicating the formation of Pd NPs [71]. Pd NPs exhibited anticancer activity (33%) against human cervical (HeLa) cancer cells [71]. Nasrollahzadeh et al. [72] reported the use of *Euphorbia granulate* leaf extract to prepare spherical Pd NPs of an average size range of 25–35 nm [72]. The nanoparticles were generated via the dropwise addition of leaf extract to Pd(II) ions while stirring at 120 °C for 5 min [72]. A characteristic absorption spectrum appeared at 260–320 nm due to the formation of dark brown colored Pd NPs [72]. The nanoparticles were stable for up to one week [72]. Gurunathan et al. [73] prepared spherical Pd NPs (5 nm) using *Evolvulus alsinoides* leaf extract as the reducing agent and investigated their anticancer activity against human ovarian cancer cells (A2780 cells) [73]. The green synthesis was conducted at 60 °C for 6 h, and the formation of Pd NPs was confirmed by the disappearance of the first peak of Pd (II) ions observed at 417 nm and the appearance of a broad absorption continuum [73]. The prepared nanoparticles were found to have significant cytotoxic activity on A2780 cancer cells at doses higher than 6 µg/mL after 24 h of incubation, suggesting their potential use in cancer therapy [73]. Spherical Pd NPs (6.36 nm) were fabricated using the leaf extract of *Filicium decipiens* at room temperature and contact duration of 2 d [74]. UV/Vis absorbance peak due to phytometabolites was observed between 650–700 nm [74]. The phytosynthesized nanoparticles were found to have pronounced bactericidal activity against both Gram-positive and Gram-negative bacteria. However, this antibacterial effect was found to be higher against *Escherichia coli* and *Pseudomonas aeruginosa* (zone of inhibition: 27 and 24 mm, respectively) in comparison to *Staphylococcus aureus* and *Bacillus subtilis* (zone of inhibition: 12 mm for both bacteria) making them promising cheap antibacterial candidates [74]. Bhakyaraj et al. [75] reported on the rapid biosynthesis of Pd NPs employing *Melia azedarach* leaf extract. The biogenic synthesis was achieved by stirring the leaf extract with the Pd (II) ions at 100 °C for 20 min. The generated spherical Pd NPs had an average size range of 10–20 nm, and their formation was confirmed by the appearance of UV/Vis absorbance peak at 280 nm, which is characteristic to the surface plasmon resonance of Pd NPs [75]. The biosynthesized Pd NPs exhibited significant bactericidal activity against Gram-positive and Gram-negative bacteria (the inhibition zones for *B. subtilis* = 8.33 ± 0.33 mm; for *S. aureus, E. coli*, *and P. vulgaris* = 7.33 ± 0.33 mm; and for *Streptococcus pneumoniae and P. aeruginosa* = 5.33 ± 0.33 mm) [75]. In addition, the Pd NPs prepared using *Melia azedarach* leaf extract showed significant larvicidal activity against larvae of *Aedes aegypti* (LC50 = 27.36%; LC90 52.50%) [75]. Two studies reported on the green synthesis of Pd NPs using *Moringa oleifera* flower and peel extracts yielding spherical nanoparticles with sizes 10–50 and 27 nm, respectively [76,77]. The first synthesis was achieved at 25 °C for 1 h [76], while the second one was completed at 300 °C for 5 min incubation employing microwave irradiation [77]. Both studies used a variety of techniques to study the prepared Pd NPs such as FT-IR, XRD, TGA, SEM, and TEM but employed the UV/Vis spectroscopy as an extra tool to confirm the production of Pd NPs [76,77]. In the first study conducted by Anand et al. [76], a strong, surface plasmon resonance was observed in the absorption spectrum at 460 nm, which indicated the formation of Pd NPs [76]. The biosynthesized Pd NPs showed anticancer activity against human lung cancer cells (A549) without inducing any toxicity toward peripheral lymphocytes normal cells. The nanoparticles also exhibited antioxidant effects and bactericidal activity against *Enterococcus faecalis* [76]. In the second study conducted by Surendra et al. [77], the phytosynthesized Pd NPs showed pronounced antibacterial activity against *Staphylococcus aureus*, and *Escherichia coli* and the inhibition zones were found out to be 0.7 mm and 0.6 mm, respectively [58]. The nanoparticles showed no toxicity (hemolysis) when tested on red blood cells [77]. Both studies support the potential of using Pd NPs mediated by *Moringa oleifera* as possible safe and effective antibacterial and anticancer therapeutic agents. 

Manikandan et al. demonstrated the synthesis of spherical Pd NPs (50–150 nm) using leaves extract of *Prunus* × *yedoensis* [78]. The eco-friendly method was conducted by the continuous stirring of Pd (II) and leaf extract in ratio 40:5 at 80 °C and pH 7 for 30 min. The appearance of a UV/Vis absorption spectrum at 421 nm indicated the formation of Pd NPs. The generated nanoparticles exhibited bactericidal activity against *Bacillus subtilis* (6 mm) and *Pseudomonas aeruginosa* (5 mm) at a concentration of 250 μg/mL [78]. A study conducted by Veisi et al. reported on the facile green synthesis of Pd NPs employing the fruit extract of *Rosa canina* [79]. The biosynthesis was carried out at 100 °C for 2 h using acetone as anti-solvent, and the average size of biosynthesized spherical Pd NPs was found to be 10 nm [79]. The disappearance of the UV/Vis absorption spectrum observed at 418 nm corresponding to Pd (II) ions suggested the reduction of Pd (II) ions and the generation of Pd NPs [79]. 

Two recent studies reported on the efficient biosynthesis of Pd NPs involving the leaves extract of the *Santalum album* [80] and *Sapium sebiferum* [81]. The Pd NPs generated using the leaf extract of *Santalum album* were prepared by mixing Pd (II) ions with the leaf extract in ratio 9:1 at room temperature for 4 d [80]. The produced spherical nanoparticles had an average size range of 10–40 nm. They showed potent antibacterial activity against Gram-negative bacteria (*E. coli*, 31 mm; *P. aeruginosa,* 30 mm) which was higher than Gram-positive bacteria (*B. subtilis*, 12 mm; *S. aureus* 18 mm) [80]. The Pd NPs produced by the leaf extract of *Sapium sebiferum* were prepared by stirring Pd (II) ions with the leaf extract in the dark at 100 °C [81]. The produced spherical nanoparticles had an average size range of 2–5 nm and exhibited significant bactericidal activity against *Staphylococcus aureus* 29 (± 0.8 mm), *Bacillus subtilis* 19 (± 0.6 mm), and *Pseudomonas aeruginosa* 11 (± 0.6 mm) [81]. The disappearance of the peak observed at 420–440 nm corresponding to Pd (II) ions suggests the formation of Pd NPs in the first study [80]. While the appearance of the surface plasmon resonance peak at 274 nm indicates the generation of Pd NPs in the second study [81]. 

A study conducted by Petla et al. reported on the phytosynthesis of spherical Pd NPs of ∼15 nm average size using protein-rich soybean leaf extract by mixing Pd (II) ionic solution with the leaf extract in ratio 20:1 for two days [82]. As for the rest of the studies reported on the green synthesis of Pd NPs using plant extracts, the generation of the Pd NPs was confirmed by the disappearance of the UV/Vis absorption spectrum observed at 420 nm corresponding to Pd (II) ions [82]. Finally, the green synthesis of Pd NPs employing the leaf extract of *Rosmarinus officinalis* was recently reported [83]. The generated Pd NPs were prepared by mixing Pd (II) ions with the leaf extract at room temperature for 24 h [83]. The produced semi-spherical and polyhedral nanoparticles had an average size range of 15–90 nm. The appearance of the standard UV/Vis absorption spectrum of Pd NPs in the range of 200–240 nm suggested the generation of Pd NPs [83]. The biosynthesized Pd NPs exhibited acceptable antibacterial activity against *Staphylococcus aureus* (46.7 mm), *E. coli* (18.0 mm), *Staphylococcus epidermidis* (39.5 mm), and *Micrococcus luteus* (41.2 mm) at a concentration of 10 µg/mL. Additionally, the created Pd NPs showed antifungal activity against *Candida parapsilosis* (19.4 mm), *Candida albicans* (8.5 mm)*, Candida glabrata* (97.1 mm)*,* and *Candida krusei* (21.8 mm) [83].

## 5. Other Biological Systems for the Pd NPs Biosynthesis

Ascorbic acid (as reducing agent) and sodium alginate (a stabilizing agent from brown algae) were mixed with the Pd (II) ions, and then heated in a microwave oven (850 W) for 3 min. The formation of Pd NPs was confirmed qualitatively by the conversion of the solution light yellow color into a dark brown color, and quantitatively by UV/Vis spectrophotometry [84]. A broad continuous absorption band at 345 nm was observed, with the disappearance of the Pd(II) ions original band at 441 nm. This indicates the bioreduction of Pd (II) ions to Pd NPs. The generated spherical Pd NPs had a size range of 13–33 nm [84]. The biosynthesized Pd NPs exhibited significant anticancer activity against A4549 lung cancer cells (48 h treatment) compared to Pd (II) acetate and cisplatin anticancer drugs. Additionally, the produced Pd NPs showed a pronounced antioxidant activity compared to Pd (II) acetate when the DPPH scavenging activity assay was conducted. The scavenging activities were 32.9 ± 3.2% and 27.2 ± 2.1% for Pd (II) acetate and Pd NPs, respectively, at a concentration of 640 µg/mL [84].

A recent study reported the green synthesis of multifunctional and spherical Pd NPs employing *Agaricus bisporus* (mushroom) fungi [85]. The NPs were generated by mixing the mushroom with Pd (II) ions in a ratio of 1:9 at room temperature until a brown color is developed, indicating the formation of Pd NPs. The formation of Pd NPs was confirmed by the disappearance of the UV/Vis absorption spectrum observed at 405 nm, corresponding to Pd (II) ions [85]. The prepared Pd NPs were stable (a zeta potential of −24.3 mV) and 13 nm in size. They exhibited significant bactericidal activity against the Gram-positive *Streptococcus pyogenes* (16 ± 1.5 mm) and the Gram-negative *Enterobacter aerogenes* (21 ± 0.5 mm) [85]. Additionally, the NPs were found to have remarkable anticancer activity against PK13 kidney cancer cells. The maximum cell growth inhibition (30%) was observed at 50 µg/mL after 36 h (IC50: 26.1 µg/mL) [85]. Furthermore, the produced NPs were found to possess antioxidant and anti-inflammatory activities and biocompatibility with red blood cells [85].

Another recent study reported on the rapid biosynthesis of Pd NPs with controllable size and shape and reduced reaction time using the urine of the indigenous Indian Khilar cow [86]. The NPs were prepared by the dropwise addition of cow urine to Pd (II) ions with continuous stirring at 80 °C and physiological pH [86]. The appearance of a UV/Vis peak at 410 nm suggests the synthesis of Pd NPs. Field emission scanning electron microscopy showed the generation of cylindrical, polydispersed Pd NPs. The biosynthesized NPs exhibited good antibacterial activities against *E. coli* (15 mm), *Bacillus cereus* (15 mm), *P. aeruginosa* (16 mm), and *S. typhi* (16 mm) at a concentration of 200 µg/mL [86]. At the same concentration, Pd NPs prepared by cow urine were found to have antifungal activity against *Aspergillus niger* (12 mm) and *Fusarium solani* (11 mm). Pd NPs also showed good antioxidant activity, compared to standard ascorbic acid, where the radical scavenging activity (using DPPH assay) was found to be 28% [86]. 

Arsiya et al. [87] created an eco-friendly and rapid procedure for the biosynthesis of stabilized, monodispersed, and spherical Pd NPs of an average size of 15 nm, employing *Chlorella vulgaris* marine algae. The marine algae were stirred with Pd (II) ions at 60 °C for 10 min and then kept in the dark [87]. The typical peak of Pd (II) ions observed at 410–420 nm disappeared after the bioreduction was accompanied by the appearance of a broad absorption continuum at a range of 370–440 nm, indicating the production of the Pd NPs [87]. Finally, the marine algae *Sargassum bovinum* was exploited for the rapid and cost-effective green synthesis of monodispersed, octahedral Pd NPs of an average size of 5 nm [88]. The marine algae were mixed with Pd (II) ions in a rotary shaker at 160 rpm for 24 h, and the temperature was kept at 60 °C. The disappearance of the absorption peaks above 300 nm, related to Pd (II) ions in the UV/Vis spectrum of Pd NPs, indicated the bioreduction of Pd (II) ions and the formation of Pd NPs. Additionally, a broad absorption band appeared and extended throughout the visible-near-ultraviolet region [88]. These findings are summarized in Table 2.

**Figure 3:**
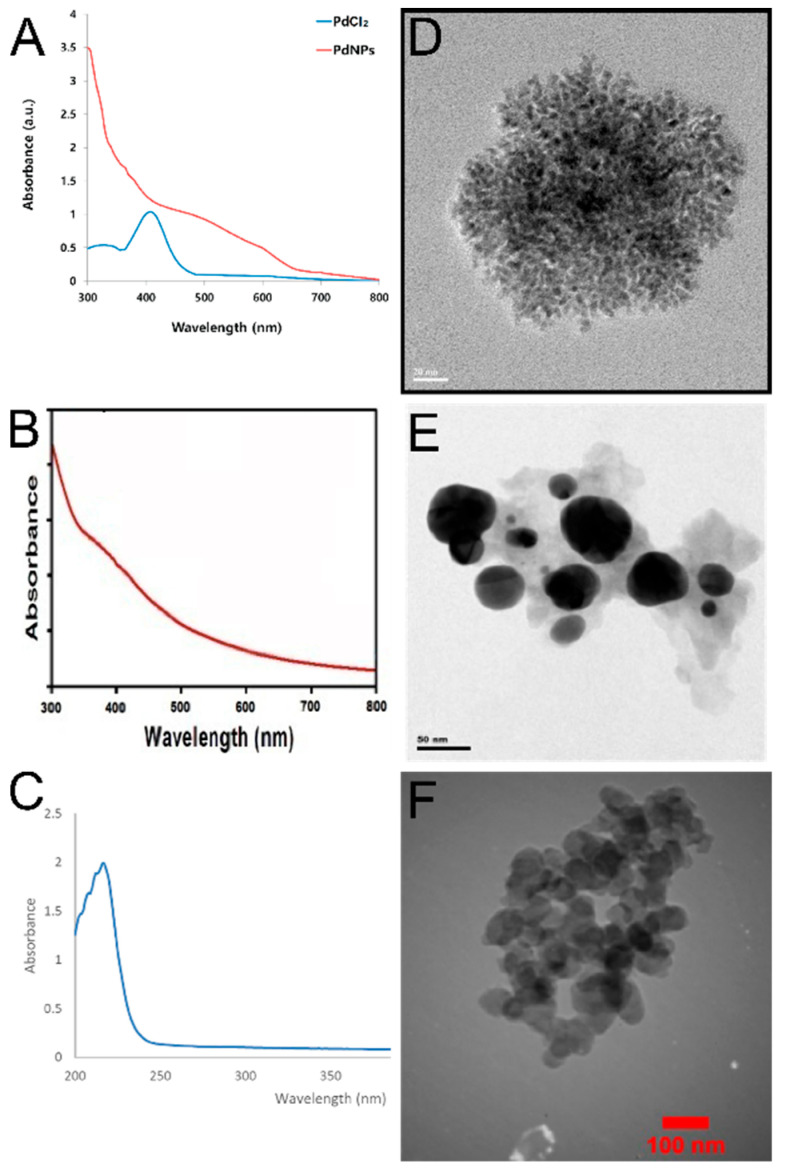
Characterization of Pd NPs biosynthesized by different plant extracts using UV/Vis spectrophotometry and TEM. (**A**) UV/Vis spectrum of Pd NPs using *Evolvulus alsinoides*, reprinted from Ref. [73]. (**B**) UV/Vis spectrum of Pd NPs using *Solanum nigrum*, with permission from Ref. [25], Copyright 2020 Elsevier. (**C**) UV/Vis spectrum of Pd NPs using *Rosmarinus officinalis*, reprinted with permission from Ref. [83], Copyright 2020 Elsevier. (**D**) TEM image of Pd NPs using *Evolvulus alsinoides*, reprinted from Ref. [73]. (**E**) TEM image of Pd NPs using *Solanum nigrum*, reprinted with permission from Ref. [25], Copyright 2020 Elsevier. (**F**) TEM image of Pd NPs using *Rosmarinus officinalis*, reprinted with permission from Ref. [83], Copyright 2020 Elsevier.

Various techniques are employed in the confirmation of Pd NPs generation, such as Fourier transform infrared spectroscopy (FT-IR), X-ray diffraction (XRD), transmission electron microscopy (TEM), scanning electron microscopy (SEM), dynamic lights scattering (DLS), and thermal gravimetric analysis (TGA) [61]. However, UV/Vis spectrophotometry and TEM provide a convenient way to confirm the production of Pd NPs (Figure 3). Table 3 summarizes the UV/Vis absorption spectra of the Pd NPs biosynthesized using different biological sources.

## 6. Biosynthesis of Pd Bimetallic NPs

Few studies reported the biosynthesis of Pd bimetallic NPs. Spherical platinum–palladium (Pt–Pd) NPs (10–25 nm) were biosynthesized using the tuber extract of *Dioscorea bulbifera* [71]. Pt–Pd NPs exhibited a remarkable anticancer activity (74%) against human cervical (HeLa) cancer cells compared to Pd NPs (33%) and Pt NPs (12.6%) alone [71].

Another study reported the green synthesis of spherical gold–palladium (Au–Pd) NPs (7 nm), using Cacumen platycladi leaf extract in aqueous medium [89]. The Au NPs exhibited a characteristic surface plasmon resonance (SPR) peak at 531 nm, while no peak was observed for the Pd NPs [89]. A study conducted by Luo et al. reported the facile biosynthesis of iron–palladium (Fe–Pd) NPs utilizing the aqueous leaf extract of grape [90]. The size range of the biosynthesized quasi-spherical Au–Pd NPs was 10–100 nm [90]. A recent study reported the green synthesis of spherical silver–palladium (Ag–Pd) NPs employing aqueous fruit extract of *Terminalia chebula* [91]. The bimetallic NPs were generated by the dropwise addition of the fruit extract to silver nitrate solution followed by stirring for 10 min. Then, Pd (II) chloride was added, followed by stirring for 1 h at room temperature. The formation of Ag–Pd NPs was confirmed by the appearance of Ag NPs UV/Vis peak at 420 nm [91]. The prepared Pd NPs were stable (zeta potential of −14.4 mV) and were 20 nm in size. They exhibited significant bactericidal activity against Gram-positive MRSA 11 (12 mm), Gram-positive MRSA 56 (14 mm), and the Gram-negative *P. aeruginosa* (16 mm) at a concentration of 30 µg/mL [91]. Additionally, the bimetallic NPs were found to have remarkable anticancer activity against A549 cancer cells with IC50 of 48.45 µg/mL compared to cisplatin anticancer drug (IC50 18.09 µg/mL) [91]. 

The biosynthesis of Pd NPs mediated by different biological entities are influenced by factors such as temperature, reaction time, the ratio of metal ions to plant extract, and pH. These factors control not only the production process of the Pd NPs, but also their sizes, shapes, and stability. For instance, smaller Pd NPs are formed at higher pH, while larger Pd NPs are formed at lower pH. Furthermore, increasing the reaction temperature could increase the yield of Pd NPs, rate of the reaction, and surface plasmon resonance. Thus, these factors should be carefully optimized to produce optimized and therapeutically active Pd NPs.

## 7. Conclusions

Plant extracts and other biological entities have been used for the biosynthesis of Pd NPs. Biosynthesis conditions such as pH, temperature, and reaction time must be optimized as they influence the size, shape, and stability of the prepared metallic nanoparticles. Plants and other biological entities are rich in diverse metabolites, making them excellent candidates for the bioreduction of palladium salts into Pd NPs. Few studies reported synthesis of Pd NPs using algae and microbes with the aim of biomedical applications. Pd NPs mediated by the biological systems showed various biomedical applications including bactericidal activity against Gram-negative and Gram-positive bacteria, antifungal, antioxidant, and anticancer activities against various cancer cells derived from lung, cervical, ovarian, and breast cancers. Pd NPs capped with plant extracts are nontoxic and biocompatible compared to platinum-based anticancer drugs such as cisplatin, carboplatin, and oxaliplatin. Moreover, biosynthesized Pd NPs were found to possess more enhanced anticancer activities compared to other synthetic anticancer drugs. These findings may lead to the development of novel anticancer and antibacterial drugs which are safe, eco-friendly, and more effective in comparison to their synthetic counterparts. 

## Figures and Tables

**Table 1:** Biosynthesis of Pd NPs using plant extracts.

Plant	Part Used	Conditions of the Reaction (Temperature, Contact Time)	Average Size (nm)	Shape	Biomedical Properties	Reference
*Solanum nigrum*	Leaf extract	Room temperature. 10 min.	21.55	Spherical and polydispersed	Antibacterial activity against *Escherichia coli*	[25]
*Cissus quadrangularis*	Stem extract	Room temperature. 10 min.	12–26	Spherical	Antibacterial activity against *Escherichia coli*	[63]
*Aspalathus linearis*	Leaf extract	Room temperature. 30 min	12.7	spherical, triangular, rectangular, cubic, and decahedron		[64]
*Bauhinia variegata*	Bark extract	Hot centrifugation at 6000 rpm. 10–15 min.	2–9	Irregular shape (cylindrical) and highly aggregated	Potent antibacterial activity against Gram-positive bacteria (*Bacillus subtilis*) Significant antifungal activity against *Candida albicans* Potent anticancer activity against MCF-7 breast cancer cell lines	[65]
*Camellia sinensis* (Lahijan Black tea)	Leaf extract	100 °C. 1 h. Pd (II): leaf extract in ratio 10:1	7	Spherical		[66]
*Camellia sinensis* (White tea)	Powder	40 °C. 2 h.	6–18	Spherical	Antibacterial activities against Staphylococcus epidermidis and Escherichia coli Antioxidant and free radical scavenging activity More anticancer activity against human leukemia (MOLT-4) cells compared to doxorubicin and cisplatin.	[6]
*Cinnamomum zeylanicum*	Bark extract	30 °C. 72 h. Incubated (dark) in a rotary shaker at 160 rpm. pH > 5	15–20	Crystalline		[67]
*Curcuma longa*	Tuber extract	30 °C. 72 h. Incubated (dark) in a rotary shaker at 160 rpm. pH > 5	10–15	Spherical		[68]
*Cinnamomum camphora*	Leaf broth	30 °C. 12 h. Incubated (dark) in a rotary shaker at 150 rpm	3.2–6.0	Quasi-spherical and irregular		[69]
Coffee and tea extract	Powder	Room temperature.	5–100	Spherical		[70]
*Dioscorea bulbifera*	Tuber extract	100 °C. 5 h	10–25	Spherical and blunt-ended cubes	Anticancer activity against human cervical (HeLa) cancer cells Antioxidant	[71]
*Euphorbia granulate*	Leaf extract	80 °C. 5 min.	25–30			[72]
*Evolvulus alsinoides*	Leaf extract	60 °C. 6 h.	5	Spherical	Anticancer activity against human ovarian cancer A2780 cells	[73]
*Filicium decipiens*	Leaf extract	Room temperature. 4 d.	6.36	Spherical	Significant bactericidal activity against Gram-negative bacteria (*E. coli*, *P. aeruginosa*) than Gram-positive bacteria (*S. aureus*, *B. subtilis*)	[74]
*Melia azedarach*	Leaf extract	100 °C. 20 min.	10–20	Spherical	Antibacterial activity against Gram-positive and Gram-negative bacterial strains Larvicidal activity against *Aedes aegypti*	[75]
*Moringa oleifera*	Flower extract	25 °C. 1 h.	10–50	Spherical	Cytotoxic activity against human lung carcinoma cells (A549) and peripheral lymphocytes normal cells Antioxidant and free radical scavenger. Antibacterial activity against *Enterococcus faecalis*	[76]
*Moringa oleifera*	Peel extract	Microwave irradiation: 300 W. 300 °C. 5 min.	27 ± 2	Spherical	More antibacterial activity against *S. aureus* than *E. coli* Nontoxic to red blood cells (RBCs)	[77]
*Prunus* × *yedoensis*	Leaves extract	80 °C. 30 min. pH 7. Pd (II): leaf extract in ratio 40:5	50–150	Spherical	Significant antibacterial activity against *Bacillus subtilis* and *Pseudomonas aeruginosa*	[78]
*Rosa canina*	Fruit extract	100 °C. 2 h	10 ± 3	Spherical		[79]
*Santalum album*	Leaf extract	Room temperature. 4 d. Pd (II): leaf extract in ratio 9:1	10–40	Spherical	More bactericidal activity against Escherichia coli and Pseudomonas aeruginosa than Bacillus subtilis and Staphylococcus aureus.	[80]
*Sapium sebiferum*	Leaf extract	100 °C. Magnetic stirring in the dark	2–5	Spherical	Enhanced bactericidal activity against Staphylococcus aureus, Bacillus subtilis, and Pseudomonas aeruginosa	[81]
Glycine max (Soya bean)	Leaf extract	2 d. Pd (II): leaf extract in ratio 20:1	∼15	Spherical		[82]
*Rosmarinus officinalis*	Leaf extract	Room temperature. 24 h	15–90	Semi-spherical and polyhedral	Acceptable antibacterial activity against *S. aureus*, *E. coli*, *S. epidermidis*, and *M. luteus* Antifungal activity against *C. parapsilosis*, *C.albicans*, *C. glabrata*, and *C. krusei*	[83]

**Table 2:** Biosynthesis of Pd NPs using various biological entities.

Biological Entity	Type	Conditions of Synthesis (Temperature, Contact Time)	Average Size (nm)	Shape	Biomedical Properties	Ref.
Sodium alginate	Brown algae	3 min	13–33	spherical	Anticancer activity against A4549 lung cancer cells Antioxidant Antibacterial Antifungal	[84]
*Agaricus bisporus*	Mushroom (fungi)	Room temperature	13	Spherical	Anticancer activity against PK13 kidney cancer cells Bactericidal activity Antioxidant Anti-inflammatory Biocompatible to RBCs	[85]
Indian Khilar cow urine		80 °C	-	Cylindrical	Antioxidant Antibacterial Antifungal	[86]
*Chlorella vulgaris*	Marine algae	60 °C 10 min	15	Spherical		[87]
*Sargassum bovinum*	Marine algae	60 °C 24 h	5	Octahedral		[88]

**Table 3:** UV/Vis absorption spectra for biosynthesized Pd NPs.

Biological Entity	Peak Appeared	Peak Disappeared	Reference
*Solanum nigrum* leaf extract	-	at 370–440 nm *	[25]
*Cissus quadrangularis* stem extract	-	at 420 nm *	[63]
*Aspalathus linearis* leaf extract	-	at 284.5 nm **	[64]
*Bauhinia variegate* bark extract	-	at 420 nm *	[65]
*Camellia sinensis* leaves extract	-	at 400 nm*	[66]
*Camellia sinensis* (White tea) extract	broad absorption continuum	at 410 nm *	[6]
*Cinnamomum camphora* leaf broth	new broad absorption continuum in the visible-near-ultraviolet region	above 300 nm	[69]
Coffee and tea powder extracts	broad continuum in the range of 200–1200 nm	-	[70]
*Dioscorea bulbifera* tuber extract	-	at 420 nm *	[71]
*Euphorbia granulate* leaf extract	spectrum at 260–320 nm	-	[72]
*Evolvulus alsinoides* leaf extract	broad absorption continuum	at 417 nm *	[73]
*Filicium decipiens* leaf extract	-	between 650–700 ***	[74]
*Melia azedarach* leaf extract	at 280 nm	-	[75]
*Moringa oleifera* flower extract	at 460 nm	-	[76]
*Prunus* × *yedoensis* leaves extract	at 421 nm	-	[78]
*Rosa canina* fruit extract	-	at 418 nm *	[79]
*Santalum album* leaves extract	-	at 420–440 nm *	[80]
*Sapium sebiferum* leaves extract	at 274 nm	-	[81]
soybean leaf extract	-	at 420 nm *	[82]
*Rosmarinus officinalis*	in the range of 200–240 nm	-	[83]
Sodium alginate	broad continuous absorption band at 345 nm	at 441 nm *	[84]
*Agaricus bisporus* mushroom	-	at 405 nm *	[85]
*Khilar* cow urine	at 410 nm	-	[86]
*Chlorella vulgaris*	broad absorption continuum at a range of 370–440 nm	at 410–420 nm *	[87]
*Sargassum bovinum*	-	above 300 nm	[88]

* corresponding to Pd (II) ions. ** corresponding to *Aspalathus linearis* leaf extract. *** corresponding to phytometabolites.
