# Correction: Fahmy et al. Palladium Nanoparticles Fabricated by Green Chemistry: Promising Chemotherapeutic, Antioxidant and Antimicrobial Agents. *Materials* 2020, *13*, 3661

**DOI:** 10.3390/ma19081609

**Published:** 2026-04-17

**Authors:** Sherif Ashraf Fahmy, Eduard Preis, Udo Bakowsky, Hassan Mohamed El-Said Azzazy

**Affiliations:** 1 Department of Chemistry, School of Sciences & Engineering, The American University in Cairo, AUC Avenue, P.O. Box 74, New Cairo 11835, Egypt; sheriffahmy@aucegypt.edu; 2 Department of Pharmaceutics and Biopharmaceutics, University of Marburg, Robert-Koch-Str. 4, 35037 Marburg, Germany; eduard.preis@pharmazie.uni-marburg.de

In the original publication [1], there were mistakes in Figure 3E and its legend. The corrected Figure 3E, together with the corrected legend, is presented below.

The authors state that the scientific conclusions are unaffected. This correction was approved by the Academic Editor. The original publication has also been updated.

## Figures and Tables

**Figure 3:**
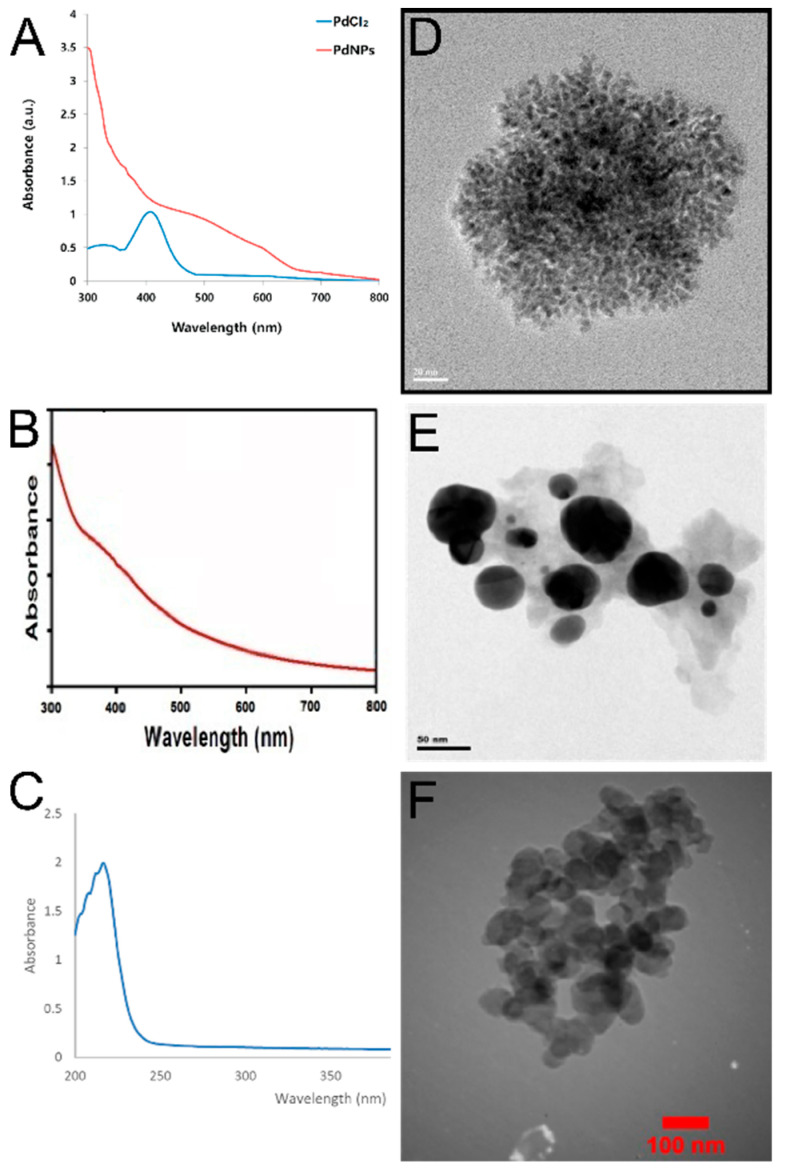
Characterization of Pd NPs biosynthesized by different plant extracts using UV/Vis spectrophotometry and TEM. (**A**) UV/Vis spectrum of Pd NPs using *Evolvulus alsinoides*, reprinted from Ref. [73]. (**B**) UV/Vis spectrum of Pd NPs using *Solanum nigrum*, with permission from Ref. [25], Copyright 2020 Elsevier. (**C**) UV/Vis spectrum of Pd NPs using *Rosmarinus officinalis*, reprinted with permission from Ref. [83], Copyright 2020 Elsevier. (**D**) TEM image of Pd NPs using *Evolvulus alsinoides*, reprinted from Ref. [73]. (**E**) TEM image of Pd NPs using *Solanum nigrum*, reprinted with permission from Ref. [25], Copyright 2020 Elsevier. (**F**) TEM image of Pd NPs using *Rosmarinus officinalis*, reprinted with permission from Ref. [83], Copyright 2020 Elsevier.
